# The Evidence for Nerve Repair in Obstetric Brachial Plexus Palsy Revisited

**DOI:** 10.1155/2014/434619

**Published:** 2014-01-16

**Authors:** Willem Pondaag, Martijn J. A. Malessy

**Affiliations:** Department of Neurosurgery (J-11), Leiden University Medical Center, P.O. Box 9600, 2300 RC Leiden, The Netherlands

## Abstract

Strong scientific validation for nerve reconstructive surgery in infants with Obstetric Brachial Plexus Palsy is lacking, as no randomized trial comparing surgical reconstruction versus conservative treatment has been performed. A systematic review of the literature was performed to identify studies that compare nerve reconstruction to conservative treatment, including neurolysis. Nine papers were identified that directly compared the two treatment modalities. Eight of these were classified as level 4 evidence and one as level 5 evidence. All nine papers were evaluated in detail to describe strong and weak points in the methodology, and the outcomes from all studies were presented. Pooling of data was not possible due to differences in patient selection for surgery and outcome measures. The general consensus is that nerve reconstruction is indicated when the result of nerve surgery is assumedly better than the expected natural recovery, when spontaneous recovery is absent or severely delayed. The papers differed in methodology on how the cut-off point to select infants for nerve reconstructive surgical therapy should be determined. The justification for nerve reconstruction is further discussed.

## 1. Introduction

At present, nerve surgical treatment is widely applied to infants with Obstetric Brachial Plexus Palsy (OBPP). Strong scientific validation of the value of nerve reconstructive surgery is lacking. Additionally, different nerve surgical treatment strategies exist amongst surgeons concerning the method and the timing to select patients for surgery.

The best guide for treatment would arise from a randomized trial comparing surgical treatment with spontaneous recovery in infants with similarly severe neurological impairment. Such a randomized trial, however, has not been performed yet [1]. Some authors strongly advocate conducting a randomized trial [1–3], but it is unlikely that this will happen in the near future [4].

The current evidence supporting nerve surgical treatment consists of comparative patient series which conclude that brachial plexus reconstruction may be beneficial in the indicated patients. The scientific methodologies in these papers differ greatly.

A systematic literature review was performed to analyse and describe the currently available comparative patient series. Special attention was given to the scientific methodology and evidence level.

## 2. Methods

A PubMed search was performed to identify papers on OBPP. The following search strategy was employed:

1. “Paralysis, Obstetric” (MESH),
2. ((Plexus (TITLE) AND Brachial (TITLE)) OR “Brachial Plexus” (MESH:noexp) OR “Brachial Plexus Neuropathies” (MESH:noexp)) AND (“Birth Injuries” (MESH) OR “Child” (MESH:noexp) OR “Infant” (MESH:noexp) OR “Infant, Newborn” (MESH:noexp)),
3. (Erb (TEXT) or Erbs (TEXT)) and (Palsy (TEXT) or Palsies (TEXT) or Paralysis (TEXT)).

The search was limited to July 07, 2013. In total 1921 publications were identified. Additionally, the reference list of key papers was investigated, and the newly identified references were added. The papers that were identified were first screened by reading title and abstract. Appropriate articles were selected for further reading. Only those papers that compared the natural history and results of nerve reconstruction in the same paper were eventually included. Nerve reconstruction by grafting and/or transfer was considered as a nerve surgical treatment. When only neurolysis was performed, this was considered as exploratory diagnostic surgery without influencing the natural course of potential neurological recovery and was, therefore, classified as conservative treatment. The raw data of the selected papers were used to construct new figures and/or tables to illustrate the findings.

The following characteristics were collected: study design (prospective/retrospective); population under analysis; number of patients in the treatment arm and conservative arm; mean age of surgery; selection strategy for surgical treatment; mean age at surgery; follow-up; method of evaluation; statistical analysis and evidence level. The level of evidence of the paper was determined using the criteria on treatment benefits of the Centre for Evidence-Based Medicine (Table 1) [5].

## 3. Results

Nine studies met our inclusion criteria of which the characteristics are summarized in Table 2. Eight series consisted of level 4 evidence, and one study [3] could be classified as level 5.

All the included studies will be discussed in more detail below and strong and weak points of the applied methodology will be outlined [3, 6–13].

### 3.1. Gilbert and Tassin 1984

The first comparison of conservative versus surgical strategy was published by Gilbert and Tassin in 1984 [6]. Both patient series had been described in more detail in Tassin's thesis [14]. For the purpose of completeness, relevant data were extracted from the thesis. The study is a comparison of two patient groups from two different hospitals. The conservatively treated group consisted of 44 conservatively treated children from hospital Saint Vincent, Paris, France, with a follow-up of five years, or until complete recovery was documented. The end stage of recovery was expressed using the Mallet scale, which is an ordinal scale to evaluate shoulder function [15]. These conservatively treated infants were compared to 38 surgically treated infants of the Hôpital Trousseau, Paris.

**Figure 1:**
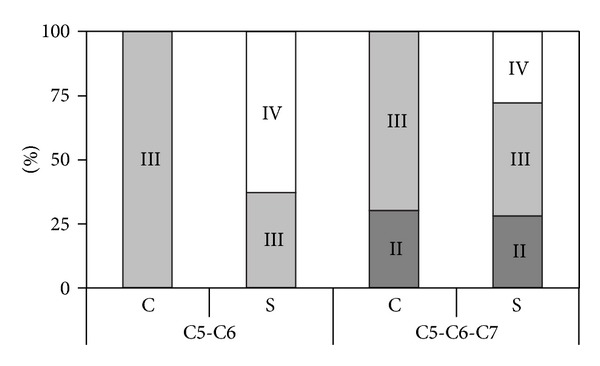
Bar diagram comparing conservative (C) with surgical (S) results. Each bar shows the percentage of patients that attain Mallet score II/III/IV. Divided into infants with C5-C6 lesions and C5-C6-C7 lesions. Reconstructed from Figure 5 in Gilbert's paper [6].

A comparison of patient groups with equal clinical picture of neurological deficit was performed. The category of C5-C6 lesions included 22 surgically treated and 18 conservatively treated patients. In the surgical group, a Mallet IV shoulder was reached in 14/22 patients (63%), while delayed spontaneous recovery showed a maximum recovery of grade III. These results are illustrated in Figure 1. A statistical analysis was not performed.

Twelve children (27% of the conservatively managed population) showed complete spontaneous recovery; it was noted that in all these children the biceps muscle had gained in strength to MRC grade 3 [16] by two months of age. In children with biceps recovery after 3 months, the end stage was incomplete. The main conclusion of this paper was that surgical treatment is warranted if the biceps muscle has not recovered at three months of age.

### 3.2. Boome and Kaye 1988

**Figure 2:**
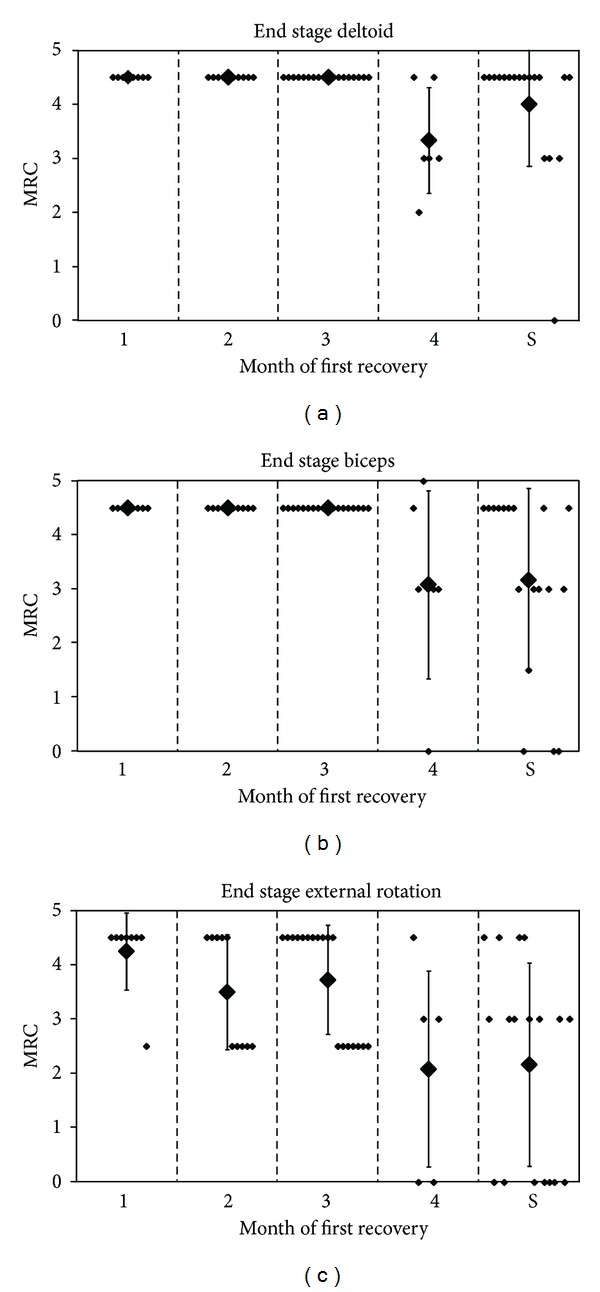
End stage of proximal functions from Boom and Kaye's data. *y*-axis: MRC score; *x*-axis: composition of groups 1 to 4 depends on the month of first recovery, compared with surgical group (S); small dots represent a patient, and large dots represent the mean score, with error bars of 1 standard deviation; graph reconstructed from Table 1 [3].

**Figure 3:**
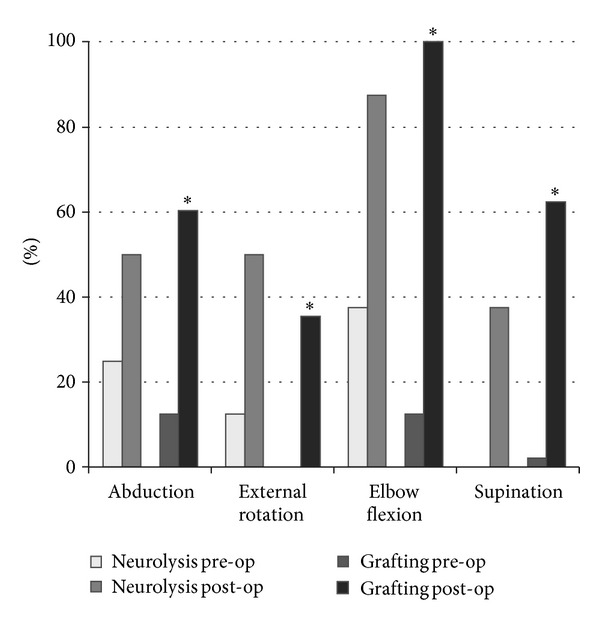
Proportion of patients with upper trunk lesions that reach a AMS score of 6 or 7. ∗ signifies statistical difference between preoperative and postoperative scores. Reconstructed from the original data in Clarke's paper [8].

Boome and Kaye performed a retrospective analysis of a group of 70 patients treated between 1981 to 1985 [3]. Twenty-two of these 70 patients underwent nerve surgery. In six of the conservatively treated infants, some follow-up data was missing. The end stage of deltoid, biceps, and external rotation function in the remaining 42 conservatively treated infants is presented in Figure 3, grouped according to the month in which the first recovery was noted. Unfortunately, the exact definition of “first recovery” was not provided.  Figure 2 is based on provided data from patient groups (spontaneous recovery) and individual patients (individual patient data) to illustrate the authors' findings.

The selection criterion for surgery was absence of both biceps and deltoid function. Of the 22 infants who underwent surgery, follow-up data were not available for two. Two patients underwent neurolysis only, and they were excluded from Figure 2.

A statistical analysis between nonoperative and operative approaches was not performed by the authors. They simply conclude from their surgical findings that spontaneous recovery would not have taken place in the surgical group. “*If recovery in the muscles innervated by the upper roots is delayed beyond three months, then root disruption is likely. Exploration and nerve grafting then offers the best prospect of a useful arm.*”

### 3.3. Clarke et al. 1998/2009

Clarke et al. analyzed the Toronto Hospital of Sick Children series in a stepwise fashion. Their first study focused on the natural history and was performed to identify specific predictors for a poor spontaneous recovery [17]. Their second study evaluated the effect of neurolysis, that is, resection of scar tissue around the nerve and occasionally the scarred outer epineurium [18]. Clarke et al. employed their own AMS system to grade muscle function: a seven-point scale was designed to express limb motion, and different joint movements were summated to form a combined test-score that was employed to set the indication for surgery.

Clarke's third study reported the outcome of graft repair of conducting neuromas in 26 patients and a cohort-like comparison was made with 16 infants from the neurolysis study (which is considered in this review as conservative treatment) [7]. The conclusion of the authors from their paper was that short follow-up (6–12 months) did not significantly diminish motor activity, which means that the conducting neuroma probably did not contain functional nerve tissue [7].

More recently, results from a larger series with minimum follow-up of 4 years were published [8]. This paper concludes that the eventual recovery after graft repair was better than after neurolysis. This conclusion was based on their finding that a recovery to AMS grade 6 or 7 was statistically more robust in the surgical repair group than in the neurolysis group. The drawback of this analysis is that a comparison is made between preoperative and postoperative AMS grading within the neurolysis and grafting groups and not a direct comparison between the end result of neurolysis versus grafting. In the grafting group the number of patients is larger, which may have led to smaller confidence intervals and greater likelihood of statistical significance.

**Figure 4:**
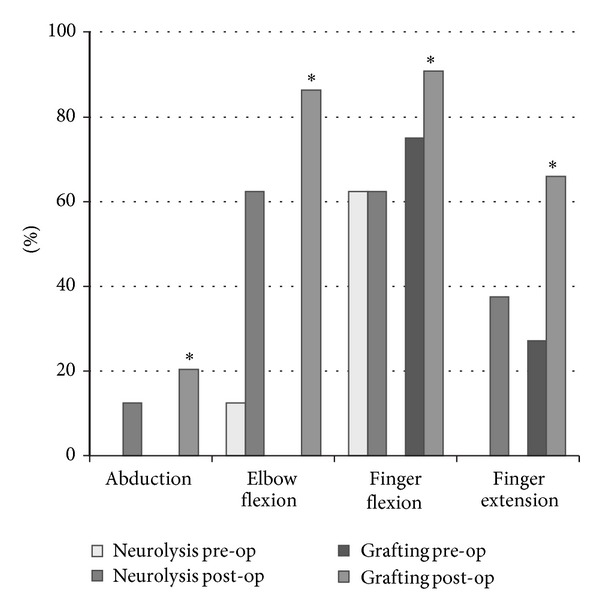
Proportion of patients with total lesions that reach a AMS score of 6 or 7. ∗ signifies statistical difference between preoperative and postoperative scores. Reconstructed from the original data in Clarke's paper [8].

Figures 3 and 4 illustrate the findings.

### 3.4. Waters 1999

**Figure 5:**
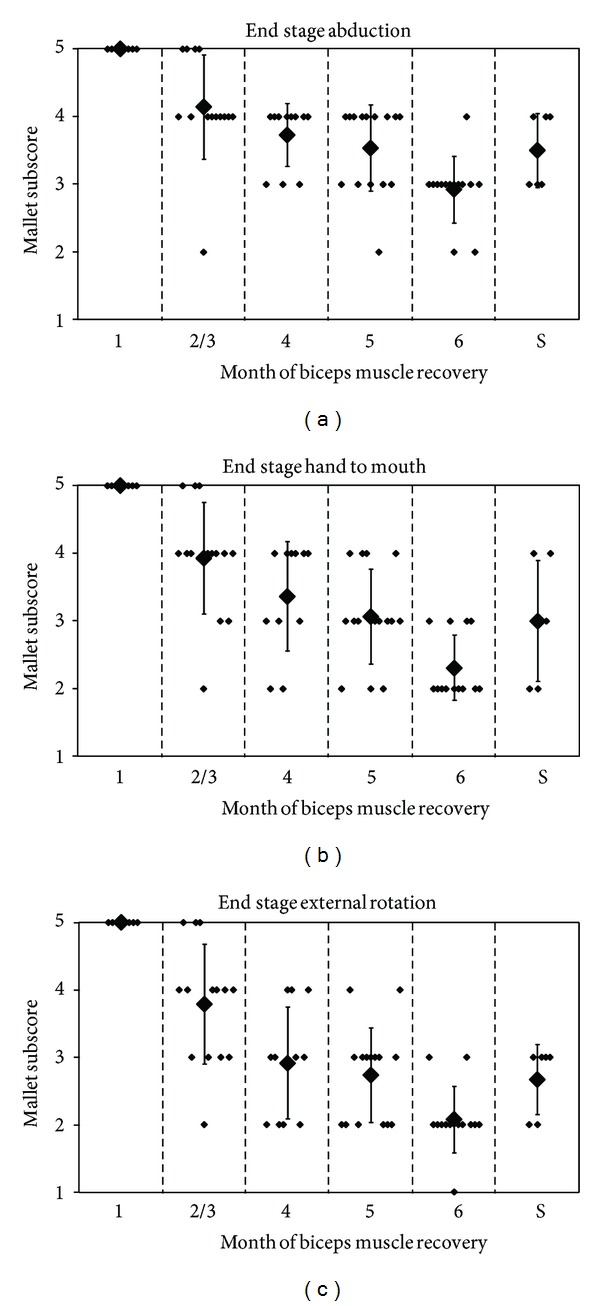
End stage of proximal functions from Waters' data. *y*-axis: Mallet subscore; composition of groups 1 to 6 depends on the month of first recovery, compared with surgical group (S); small dots represent a patient, and large dots represent the mean score, with error bars of 1 standard deviation; graph reconstructed from published individual patient data [9].

Waters described 66 patients seen in a 6-year period [9]. Of these patients, 27 had been referred after the age of six months and were, therefore, excluded from the analysis. Of the remaining 39 patients, 6 were surgically treated because of a lack of recovery of biceps function at the age of six months. The other children were divided into five groups, according to the month in which the biceps muscle recovered. Due to small numbers, the second and third months were pooled. Four of the five movements of the Mallet scale were analysed separately (abduction, external rotation, and the ability to bring the hand to the mouth and to the neck) instead of a composite score. It was concluded that recovery after nerve repair was better than the conservatively treated group of children with recovery of biceps function in the fifth month, but not better compared to the group that recovered in the fourth month.  Figure 5 illustrates the different end stages in each group.

The three following graphs were derived from the original data. Unfortunately, the statistical method used for comparison of groups was not mentioned. A particular weakness of the analysis is that the late referred children (27 of 66) were excluded from analysis.

However, three important conclusions can be drawn from this study. The first is that early recovery (before one month of age) results in complete spontaneous recovery. Second, when recovery starts at four to five months, functional impairment at the end stage remains. It is, however, uncertain that a better outcome could have been achieved with nerve surgery because surgery was only decided after six months. Only when biceps recovery was delayed until the sixth month did nerve surgery after six months yield superior results. A third conclusion is that external rotation showed poorer recovery than abduction, whether spontaneously or as a result of suprascapular nerve grafting.

### 3.5. Al-Qattan 2000

In this study, the Toronto AMS outcome scale was combined with Gilbert and Waters' criterion of isolated elbow flexion recovery [10]. Al-Qattan described the results of 43 children selected from 160 cases seen over a 5-year period, excluding late referrals and incomplete follow-up. This might have created an inclusion bias. The children were divided into 4 groups, according to the month in which “active” elbow flexion started. Unfortunately a clear definition of what was considered active elbow flexion was not provided. Al-Qattans findings are summarized in Table 3.

It was concluded that with early recovery of elbow flexion, good spontaneous recovery can be expected, but when it starts after 4 months, about half of the infants will have a significant residual deformity at the level of shoulder movement.

**Figure 6:**
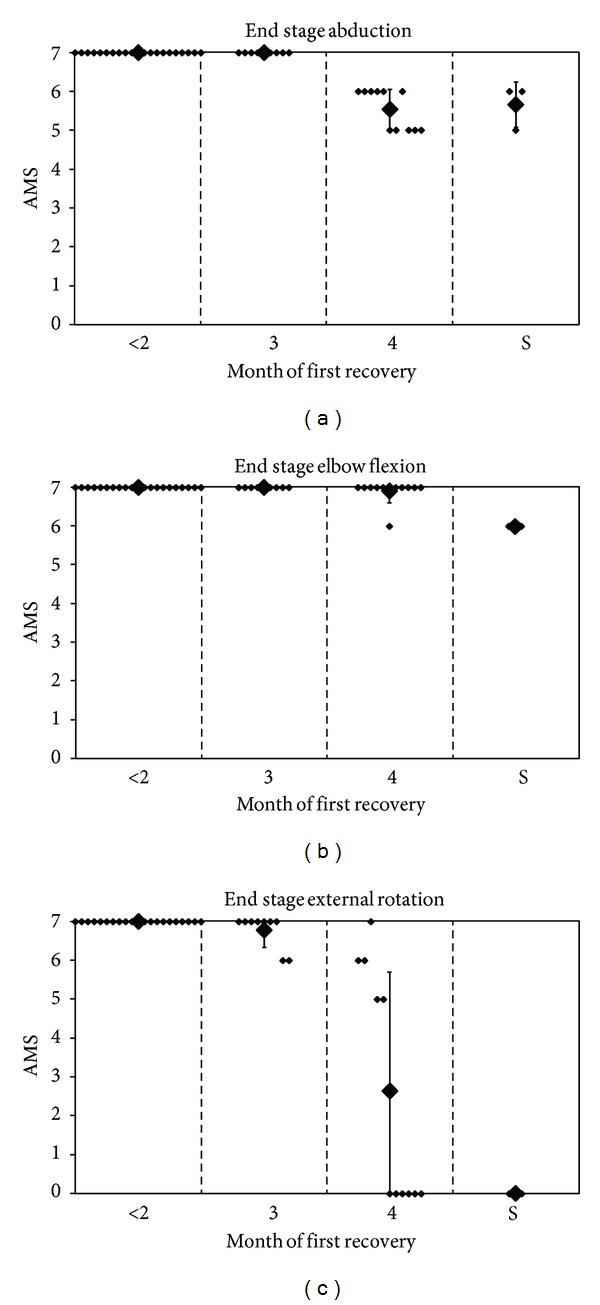
End stage of proximal functions from Al-Qattan's data. *y*-axis: active movement scale; composition of groups 2 to 4 depends on the month of first recovery, compared with surgical group (S); small dots represent a patient, and large dots represent the mean score, with error bars of 1 standard deviation; graph reconstructed from published individual patient data [10].

Only a small number of children were eventually nerve-surgically treated (*n* = 3). Therefore, a proper comparison between treatment arms cannot be performed. Just like in Waters' paper, it was shown that delayed recovery of the biceps muscle mainly has an effect on poorer spontaneous recovery of external rotation. The corresponding results are illustrated in Figure 6.

### 3.6. Xu et al. 2000

Xu et al. reported 31 patients from Fujian, China [11]. Twelve of these were treated conservatively in other hospitals for 3 to 4 years. In this group, delayed biceps recovery had been documented as occurring 5 to 8 months after birth. The remainder of the children were operated by the author because they had no recovery of biceps function by 3 months of age. In the first nine children (treated between September 1994 and May 1995), the procedure was limited to neurolysis because a conducting neuroma (based on direct stimulation of C5 and C6 and needle recording in the related muscles) was found during surgery. Ten subsequent children were treated by nerve transfer and grafting between May 1995 and June 1996. The composition of the study groups is provided in Table 4.

**Figure 7:**
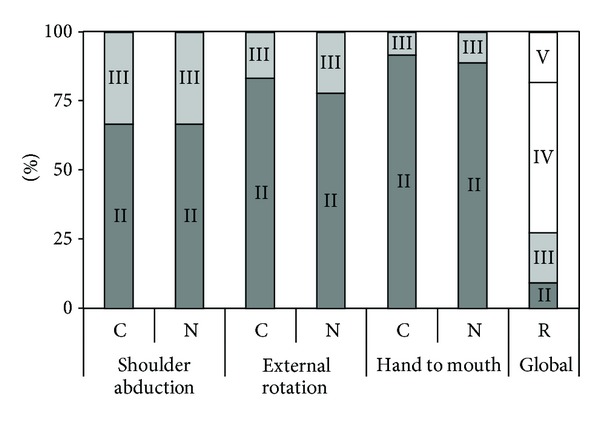
Bar diagram comparing conservative therapy (C) with neurolysis (N) and reconstruction (R). Each bar shows the percentage of patients that attain Mallet score II/III/IV/V; for the C and N groups Mallet subscores for abduction, external rotation, and hand to mouth were available; for the R group only a global Mallet score was available; reconstructed from Xu's data [11].

The shoulder and elbow functions of 12 children in the conservative group and nine children in the neurolysis group were all evaluated as being Mallet II or III; none achieved Mallet IV. No statistical difference was found between the conservative and neurolysis group. In contrast, two out of 10 patients in the nerve transfer and grafting group achieved a full recovery of shoulder and elbow motion range, and five patients reached a Mallet IV grading (Figure 7).

There are two shortcomings in this paper. First, a selection bias was introduced, as the referred patients in the conservative group came from other hospitals. Secondly, there was a difference in the evaluation measure used for the neurolysis group and the reconstruction group: for the neurolysis groups, Mallet subscores were presented, and for the reconstruction group the global Mallet score was provided.

### 3.7. Strömbeck et al. 2000

Strömbeck et al. presented children who were referred to a national OBPP clinic in Stockholm, Sweden [12]. Only those with a follow-up of more than five years were selected for analysis: 247 of a total cohort of 470. More recently a follow-up study was published [19, 20].

These 247 children were analyzed in great detail. Movements were scored according to their own scoring system: 0 (no movement), 1 (<50% ROM), 2 (>50% ROM but not full range), and 3 (normal movement). For each joint, a number of parameters were measured and added to produce a sum score. In the shoulder joint, five different parameters were measured (extension, flexion, abduction, internal rotation, and external rotation) resulting in a maximum score of 15. The additional protocol included tactile sensibility, pick-up test, grip-test, grip, bimanual activity, and hand preference. The children who “*exhibited some muscle activity in their biceps or deltoid muscles at the first visit at 3 months of age”* were considered to have early recovery (ER). A statistical analysis was performed to detect differences among treatment groups. The composition of the study groups is presented in Table 5.

**Figure 8:**
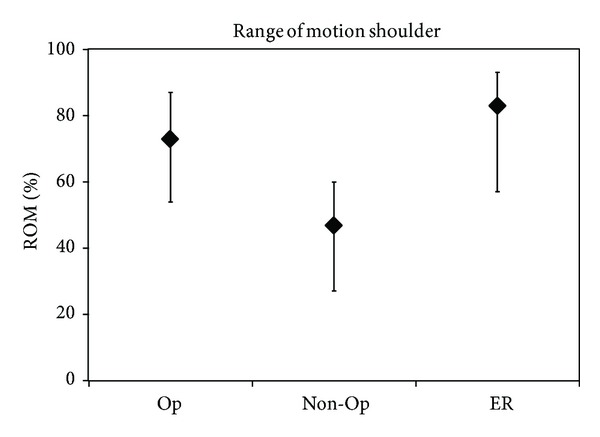
Recovery of shoulder movements. *y*-axis: attained result as percentage of the maximum score of range of motion (ROM); the median value is depicted as well as the 25th–75th percentiles; redrawn from Strömbeck's Figure 3(a) [12].

The authors concluded that all children with complete recovery by 5 years had regained “some activity” before 2 months of age. “Some activity” was unfortunately not defined clearly. Second, in the C5-C6 group, children who had undergone surgery did better than the nonoperative delayed recovery group, as far as shoulder movements were concerned (Figure 8). There was no difference in elbow flexion in this group.

**Figure 9:**
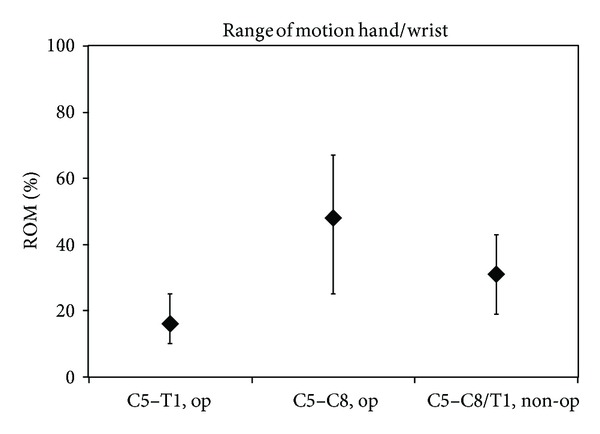
Recovery of distal movements. *y*-axis: attained results as percentage of the maximum score of range of motion (ROM); the median value is depicted as well as the 25th–75th percentiles; redrawn from Strömbeck's Figure 5(c) [12].

In children with a C5–C7 lesion, there was no difference in shoulder or elbow motion between the late recovery and operated group, while both did worse than the early recovery group. Children with a C5–C8 or C5-T1 lesion had severely diminished shoulder function, elbow flexion, and hand function. The authors could not detect statistical differences in the outcome, apart from the unsurprising finding that infants with an intact T1 root did better (Figure 9).

Despite the rigorous and extensive examination of all children, it is difficult to properly compare natural recovery and surgical results. One of the difficulties is the authors' use of a novel scoring system in which sum-scores of multiple movements were examined. Such a sum-score is difficult to relate to the clinical picture.

The major limitation is the inconsistent selection criterion for surgery, which is also acknowledged as such in the paper. Initially, it was planned that all infants with C5-C6 and C5–C7 lesions who had no biceps function at 3 months of age would be candidates for surgery. While waiting for the operation, some children unexpectedly gained good biceps function. As a consequence, the indication for surgery was delayed until the infant was 6 months or older. Additionally, several parents of children not selected for operation wanted their child to have surgery and vice versa. All 33 children with C5–8 (Th1) lesions were recommended to have an operation at the first visit at 3 to 6 months of age. Six were eventually not operated: five because they were considered too old (>18 months) for the operation at the first examination and one because of co-morbidity. In Table 6 the age groups are presented.

### 3.8. Badr et al. 2009

A more conservative approach was presented from Louisiana, USA [13]. A series of 169 patients (with 171 palsies) referred to a specialized center was presented. Only 16 children were surgically treated (9%), and the authors conclude that by using this selective approach, good outcomes were obtained, as determined by biceps and shoulder abduction grading, both in those children who did not have surgery and those who underwent surgical intervention. Only the very severe cases were surgically treated, at a mean age of 18 months (series from 1975 to 1991) and 14 months (from 1991 to 2003).

Unfortunately, the authors used impairment rating (IR) as outcome system, which makes this series difficult to compare to other series. An IR of 1 represents “almost no abnormality,” and an IR of 2 represents “slightly weak shoulder depressors, elbow flexion (antigravity), and good hand function.” Starting from an IR 3 (“shoulder abduction <90 degrees, elbow flexion—not antigravity, waiter's tip posture, and good hand function”) results could be interpreted as fair.

**Figure 10:**
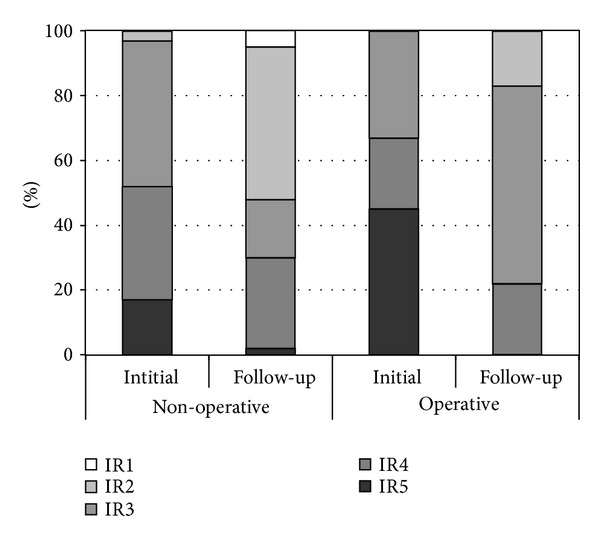
Results from the Louisiana series (*n* = 151 from 1995 to 2001) [13].

The results from 151/171 palsies with complete records including scoring of the IR show, however, that in the non-operative group only 52% recover to good or excellent (IR1 or IR2). These fair results might represent a referral pattern of patients with severe lesions but, however, stratification for lesion severity was not provided. The end stage are illustrated in Figure 10.

## 4. Discussion

The main conclusion of the present review is that the methodological quality of papers supporting the surgical treatment of OBPP differs greatly. All studies qualified as observational studies, mostly case-series, historically controlled study, and poor quality cohort studies, and provide only level 4 evidence.

Unfortunately, it is impossible to properly summarize the discussed studies. The first problem in all studies is the referral bias and inclusion bias. This obviously results in difficulties with extrapolation of the findings to the complete population of OBPP infants.

The second problem is that all these papers use different outcome measures, each with its own limitations; hence, pooling of data is not possible. The third problem is that statistical analysis is sometimes not performed, and numbers are generally small. No study carried out a power analysis. The fourth drawback of these papers is publication bias: proponents of surgical therapy may be more likely to publish on the merits of surgical intervention.

Two papers could not be included in the present analysis, because a direct comparison between the surgically treated children and the conservatively treated children could not be distilled from the data in the paper. The authors have a more reluctant approach to nerve reconstruction, especially for upper trunk lesions [21, 22]. Preoperative or intraoperative electrophysiology was used for the decision to perform nerve reconstruction or not. In both series, recovery of the biceps muscle is very good; however, a substantial number of patients (27%) needed secondary surgery for the shoulder. This can be interpreted as poor recovery of shoulder function, especially external rotation [23, 24].

The key approach of modern evidence-based medicine is that depending on the quality of the applied methodology, the level of evidence is determined, which subsequently leads to a specified grade of recommendation. The highest level of evidence (1A) is provided by a systematic review of randomized trials [5]. Particularly in surgical disciplines, however, difficulties in randomized controlled trials were outlined as follows: equipoise (both patients and surgeons), bias (selection and observer), blinding, learning curve, effectiveness versus efficacy, and standardization of technique [25].

Although nerve repair in OBPP has not been investigated in a randomized fashion compared with the natural recovery, and the evidence supporting surgical treatment is of low quality, it would be erroneous to conclude that there is no place for surgical treatment.

The main justification of nerve surgery is formed by the poor outcome of spontaneous recovery in a certain percentage of patients. This is demonstrated by the following findings.

1. Around 30% of infants with OBPP do not show complete spontaneous recovery [26] which has a permanent impact on daily life.
2. For infants with a total lesion, without any functional recovery at one to two months (frequently accompanied by Horner's syndrome), the prospect is poor. Both in historical papers [27] and in a more recent paper [28], it is concluded that spontaneous recovery of useful hand function does not occur.
3. Autopsies [29] and surgical exploration [3, 6] revealed totally ruptured nerves and nerves avulsed from the spinal cord. Such findings of complete discontinuity of the peripheral nerve exclude any spontaneous recovery in this particular nerve.

In severe lesions, with avulsions and ruptures, the neurological prognosis without treatment is very poor. This justifies intervention to improve prognosis in this group. Nerve reconstruction was shown to lead to neurological recovery even for hand function [8, 30–32]. For most physicians caring for infants with OBPP, a severe lesion with a diminished hand function without speedy recovery is a strong indication for surgical intervention. It is common for such severe lesions to consist of root avulsions at one or more levels [33]. This subgroup of patients only contains, however, about 15% of patients.

The most difficult group to select for surgery is those children who present with a C5-C6 or C5-C6-partial C7 lesion. Decision-making in this patient group was poetically called “the gray zone” [34]. The anatomical substrate of these OBPPs is usually a neuroma-in-continuity of the superior trunk. In such lesions, the damaged nerve tissue serves as a bridge for impaired, disorganized axonal outgrowth. Depending on the anatomical integrity of the different parts of the nerve elements involved, varying grades of recovery may take place. This may lead to axonal continuity in some way between the proximal and distal stump, which was demonstrated by electrical conduction studies [35, 36] and by histological investigation [37] of the neuroma. However, the extent to which this partial axonal continuity leads to clinical recovery at the end stage is not known. A gold standard for documenting spontaneous recovery of such lesions for use as comparison with surgical results is not available and probably cannot be established [4].

From the included papers that stratify the outcome based on the evolution in time [3, 6, 9, 10] it may be concluded that recovery of the biceps before the third month is a predictor of complete or near complete recovery of shoulder function. Additionally, with the increasing delay of initial recovery, the prospect of a good eventual outcome decreases. The exact cut-off point to perform nerve reconstruction cannot be determined but is probable somewhere between the 3rd and 6th month. It is important to realize that starting recovery of the biceps muscle is employed as a proxy for prognostication of the shoulder in upper trunk lesions and not per se a predictor for the end stage of recovery of the biceps muscle itself. In our experience, and that of others [21, 23], it is very seldom that conservatively treated children do not recover elbow flexion spontaneously, but in these children major deficits may remain in shoulder function.

An alternative to nerve surgical treatment of OBPP is to await the degree of natural neurological recovery and to treat residual deficits with muscle/tendon transfers, rotation osteotomy, or joint fusion. This strategy has two limitations. First, a functioning muscle must be available for transfer. If the initial nerve lesion consists of a flail arm, no functioning muscle is available to restore hand function. Thus, only the upper trunk lesions are suitable for this approach. Second, secondary surgery can be employed as an additional procedure should neurological recovery be incomplete after nerve reconstruction. Performing nerve reconstruction after a failed muscle transfer is not possible.

Moreover, it does not seem logical to leave a nerve lesion which is repairable in place to wait for recovery that is not likely to occur and to perform an orthopaedic salvage procedure at a later stage.

## 5. Conclusion

The current literature that supports surgical treatment of OBPP is formed by observational studies of patient series that compare surgical to conservative therapy. The methodological quality of these papers differs greatly but is essentially grade 4 evidence. The major drawback is that none of the individual studies provides enough scientific proof that nerve reconstruction is superior to conservative treatment and that pooling of the results is not possible due to different methodology.

The shared conclusion of most papers is that good functional results of nerve reconstruction have been accomplished in children in whom no substantial improvement is expected from conservative treatment. In our view this leads to the conclusion that a well-selected group of patients that show no recovery or delayed recovery probably benefits from nerve surgery. As the selection process for surgery differs between the studies, a definite conclusion on how to select patients for surgery cannot be drawn.

## Figures and Tables

**Table 1:** Levels of evidence.

Level	Criteria
1	Systematic review of randomized trials or n-of-1 trials
2	Randomized trial or observational study with dramatic effect
3	Nonrandomized controlled cohort/follow-up study
4	Case-series, case-control studies, or historically controlled studies
5	Mechanism-based reasoning

**Table 2:** Summary of included papers.

Paper	Design	Population	CT (*n*)	NR (*n*)	Selection	Mean age of surgery	Follow-up	Evaluation	Statistics	Evidence
Gilbert and Tassin 1984 [6]	Retro	Comparison of referred patients to two different hospitals with different treatment	44	38	Biceps strength at 3 mo			Mallet	None	4

Boome and Kaye 1988 [3]	Retro	Patients selected and not selected for surgery	42 CT + 2 neurolysis	18	Biceps and deltoid strength	5.3 mo	18 mo (1 mo–5 yr)	MRC	None	4/5

Capek et al. 1998 [7]	Retro*	Patients with nerve reconstruction compared to (historical) cohort of neurolysis	16 neurolysis	26	AMS composite score	9.0 mo (±2.3)	12 mo	AMS	Yes	4

Lin et al. 2009 [8]	Retro*	Patients with nerve reconstruction compared to (historical) cohort of neurolysis	16 neurolysis	92	AMS composite score	7.8 mo (±2.7)	min 4 yr	AMS	Yes	4

Waters 1999 [9]	Retro	Patients selected and not selected for surgery.	33	6	Biceps strength at 6 mo		2–11 yr	Mallet	Yes	4

Al-Qattan 2000 [10]	Retro	Patients selected and not selected for surgery.	30	3	Biceps strength at 4 mo		min 18 mo	AMS	Yes	4

Xu et al. 2000 [11]	Retro	Late referrals treated conservatively versus neurolysis versus reconstruction	12 CT + 9 neurolysis	10	Biceps strength at 3 mo	5 mo (3–6 mo)	40–54 mo	Mallet	Yes	4

Strömbeck et al. 2000 [12]	Retro	Patients selected and not selected for surgery excluding early recovery	53	59	Inconsistent: biceps strength at 3 or 6 mo	3–12 mo	min 5 yr	Own scoring system	Yes	4

Badr et al. 2009 [13]	Retro	Patients selected and not selected for surgery	155	16	No biceps or shoulder function at 7–10 mo	16 mo (8–36 mo)	24 mo	Impairment rating	None	4

CT: conservative treatment; NR: nerve reconstruction; Retro: retrospective; *retrospective analysis of prospectively collected data; mo: month(s); yr: year(s); min: minimal.

**Table 3:** Residual deformity depending on the month of biceps recovery from Al-Qattan's data [10].

Biceps recovery (months)	*n *	Complete spontaneous recovery	Mild residual deformity	Significant residual deformity	Poor spontaneous recovery
<2	20	20			
At 3	9	6	3		
At 4	11		5	6	
Not at 5	3				3

The group with poor spontaneous recovery was nerve-surgically treated.

**Table 4:** Composition of Xu's study groups [11].

	Total	Extent of lesion		
	*n *	C5-C6	C5–C7	C5-T1
Conservative	12	5	3	4
Neurolysis	9	3	4	2
Reconstruction	10	4	4	2

**Table 5:** Composition of Strömbeck's study groups [12].

	Total	Lesion			
	*n *	C5-C6	C5–C7	C5–C8	C5-T1
ER	135	106	29	—	
non-Op	53	15	32	6	
Op	59	8	24	8	19

Total	247	129	85	33	

Groups: ER: early recovery; non-Op: nonoperative; Op: operative.

**Table 6:** Timing of surgery stratified by lesion severity [12].

	<6 mo	7–12 mo	>12 mo
C5-C6 (-C7)	5	22	5
C5–C8 (-T1)	12	14	1
